# The Serum S100B Level as a Biomarker of Enteroglial Activation in Patients with Ulcerative Colitis

**DOI:** 10.1155/2014/986525

**Published:** 2014-03-30

**Authors:** Asuman Celikbilek, Mehmet Celikbilek, Seda Sabah, Nermin Tanık, Elif Borekci, Serkan Dogan, Yavuz Akin, Suleyman Baldane, Kemal Deniz, Neziha Yilmaz, Omer Ozbakir, Mehmet Yucesoy

**Affiliations:** ^1^Department of Neurology, Medical School, Bozok University, 66200 Yozgat, Turkey; ^2^Department of Gastroenterology, Medical School, Bozok University, 66200 Yozgat, Turkey; ^3^Department of Medical Biology, Medical School, Bozok University, 66200 Yozgat, Turkey; ^4^Department of Internal Medicine, Medical School, Bozok University, 66200 Yozgat, Turkey; ^5^Department of Gastroenterology, Medical School, Erciyes University, 38039 Kayseri, Turkey; ^6^Department of Internal Medicine, Medical School, Erciyes University, 38039 Kayseri, Turkey; ^7^Department of Pathology, Medical School, Erciyes University, 38039 Kayseri, Turkey; ^8^Department of Infectious Diseases and Microbiology, Medical School, Bozok University, 66200 Yozgat, Turkey

## Abstract

*Objective*. Recent studies have demonstrated that enteric glial cells (EGC) participate in the homeostasis of the gastrointestinal tract. This study investigated whether enteroglial markers, including S100B protein and glial fibrillary acidic protein (GFAP), can serve as noninvasive indicators of EGC activation and disease activity in UC patients. *Methods*. This clinical prospective study included 35 patients with UC and 40 age- and sex-matched controls. The diagnosis of UC was based on standard clinical, radiological, endoscopic, and histological criteria. Clinical disease activity was evaluated using the Modified Truelove-Witts Severity Index. Serum samples were analyzed for human GFAP and S100B using commercial enzyme-linked immunosorbent assay kits. *Results*. GFAP was not detected in the serum of either UC patients or controls (*P* > 0.05). However, we found a significant (*P* < 0.001) decrease in the serum S100B levels in the UC patients. No correlation between the serum S100B level and the disease activity or duration was observed (*P* > 0.05). The serum S100B levels did not differ between UC patients with active disease (24 patients, 68.6%) or in remission (11 patients, 31.4%) (*P* > 0.05). *Conclusions*. Ulcerative colitis patients had significantly lower serum S100B levels, while GFAP was of no diagnostic value in UC patients.

## 1. Introduction

Evidence is accumulating that enteric glial cells (EGC) are the morphological and functional equivalent of the astrocytes found in the central nervous system (CNS) [1].Recent studies have demonstrated that EGC participate in homeostasis of the gastrointestinal tract [2]. In transgenic mouse models in which EGC are selectively ablated, the loss of glia results in intestinal inflammation and disruption of the epithelial barrier [3].* In vitro* experiments showed that chronic EGC activation causes the release of proinflammatory cytokines and nitric oxide (NO) in the enteric neuroimmune network [4, 5]. NO is a weak radical produced from l-arginine via the enzyme NO synthase (NOS). Because increased NO production via inducible NOS (iNOS) is involved in the pathogenesis of inflammatory bowel disease (IBD) [6], alterations in enteroglial structure have been studied extensively in patients with IBD [7–9].

Demonstration of the increased enteroglial-derived S100B protein expression together with a significant increase in NO production in intestinal biopsy specimens of patients with celiac disease and ulcerative colitis (UC) [10] suggests that EGC participate directly in chronic mucosal inflammation via S100B upregulation stimulating NO production. These findings were confirmed using* in vitro* cultures of human EGC exposed to proinflammatory stimuli, in which significant increases in the cell proliferation rate, expression of S100B protein, and NO production consequent to the induction of EGC-derived iNOS protein were seen when EGC are activated [4, 5].

Enteric glial cells express not only S100B, but also high levels of glial fibrillary acidic protein (GFAP) [7]. Several studies have shown that S100B immunoreactivity mostly colocalizes with the GFAP-positive enteroglial mucosal network in tissue specimens from patients with intestinal inflammation [1, 5]. Similarly, in the CNS, astrocytes induce endothelial cells to form the tight junctions of the blood-brain barrier (BBB) [7]. A peripheral blood-tissue barrier similar to the BBB exists in the intestine where the neuronal plexi are similarly impermeable to systemic macromolecules [7]. In this sense, EGC, besides releasing cytokines, likely control the integrity and permeability of the submucosal blood vessels to confer intestinal epithelial barrier functions, as demonstrated in animal studies in which ablation of the enteroglial network enhances intestinal vascular permeability, resulting in severe mucosal inflammation [3, 7]. CNS astrocytic glial markers have been studied extensively in the peripheral blood of patients with either traumatic brain injury [11] or cerebral infarcts [12]. Although suggestive, data on EGC-derived glial markers are limited to the abovementioned experimental models of intestinal inflammation in the human gut [1, 4, 5, 10], while there is a lack of data on serum levels. We hypothesized that a disruption of EGC populations could lead to altered serum levels of EGC-derived S100B and GFAP via disruption of the peripheral blood-tissue barrier in patients with UC. This study investigated whether these enteroglial markers could serve as noninvasive indicators of EGC activation and the disease activity in patients with UC.

## 2. Methods

### 2.1. Study Population

Thirty-five UC patients from Erciyes University Medical School, Gastroenterology Department, and 40 age- and sex-matched controls from Bozok University Medical Faculty, Neurology Department, ranging in age from 30 to 65 years, were enrolled in this cross-sectional prospective study between January 2012 and June 2013. The diagnosis of UC was based on standard clinical, radiological, endoscopic, and histological criteria. Clinical disease activity was evaluated using the Modified Truelove-Witts Severity Index (MTWSI) [13–15]. Clinical active disease was defined as an estimated MTWSI score of 4 or higher and patients with a score lower than 4 were considered to be in remission (inactive). The UC patients included were all on mesalazine, and 11 (31.4%) were in remission with immunosuppressive agents including azathioprine or steroids. Patients with malignancy, chronic renal, hepatic, cardiovascular, or connective tissue diseases, thyroid disease, diabetes mellitus, chronic obstructive pulmonary disease, a history of local trauma or surgery, and acute or chronic infections were excluded from the study. In addition, those who were pregnant, morbidly obese, current smokers, or current consumers of alcohol were excluded. The study protocol was approved by the Bozok University Research Ethics Committee, and written informed consent was obtained from all participants. Body mass index (BMI) was calculated as the weight in kilograms divided by the square of height in meters [16]. Fasting venous blood samples were analyzed for measuring the erythrocyte sedimentation rate (ESR), C-reactive protein (CRP), and white blood cell count (WBC) using standard methods.

### 2.2. Serum Collection

Serum specimens were collected from all participants at the first medical examination. The specimens were centrifuged at 5000 rpm for 5 min, after which the supernatant was removed immediately and kept frozen at −80°C until assayed. All serum samples were prepared within 30 min.

### 2.3. Serum GFAP and S100B Assays

Serum samples were analyzed for human GFAP and S100B using commercial enzyme-linked immunosorbent assay (ELISA) kits for human GFAP and S100B (BioVendor Research and Diagnostic Products; Heidelberg; Germany). The samples were assayed following the manufacturer's protocol in the laboratory of Bozok University Medical Faculty.

For the GFAP ELISA, 100 *μ*L of diluted standard, quality controls, and serum samples were applied to an antibody-coated microtiter plate and incubated for 120 min, with shaking at 300 rpm on an orbital microplate shaker. The plate was washed three times with 0.35 mL of wash solution. After washing, 100 *μ*L of biotin labeled antibody solution was added to each well and incubated for 60 min, with shaking at 300 rpm. After washing, 100 *μ*L of conjugate was added and the plate was incubated for 60 min, with shaking at 300 rpm. After the third wash, 100 *μ*L of substrate solution was added and the plate incubated for a further 15 min in the dark. Then, 100 *μ*L of stop solution was added and the absorbance was measured at 450 nm on a microtiter plate reader. The GFAP levels in the culture medium were determined using a standard curve of GFAP. Similarly, serum samples were used for S100B ELISA.

To minimize the assay variation, all serum samples were analyzed on the same day in the same laboratory batch and by the same analyst at Bozok University Medical Faculty. The limits of detection for human GFAP and S100B were 0.045 ng/mL and 15 pg/mL, respectively. The serum GFAP concentrations are expressed in ng/mL, whereas the S100B concentrations are expressed in pg/mL.

### 2.4. Statistical Analysis

The Shapiro-Wilk test, histograms, and q-q plots were used to test the normality of the data, and Levene's test was used to assess the variance in homogeneity. Independent-sample* t*-tests and Mann-Whitney* U* tests were used to compare differences between continuous variables, and chi-square (*χ* ^2^) analyses were used to assess differences between categorical variables. Pearson correlations were used to examine the relationships between S100B and UC disease activity and duration. Values are expressed as frequencies and percentages, means and standard deviations, or medians and interquartile ranges. Analyses were conducted using SPSS 15.0 (SPSS; Chicago, IL; USA), and statistical significance was set at *P* < 0.05.

## 3. Results

**Figure 1:**
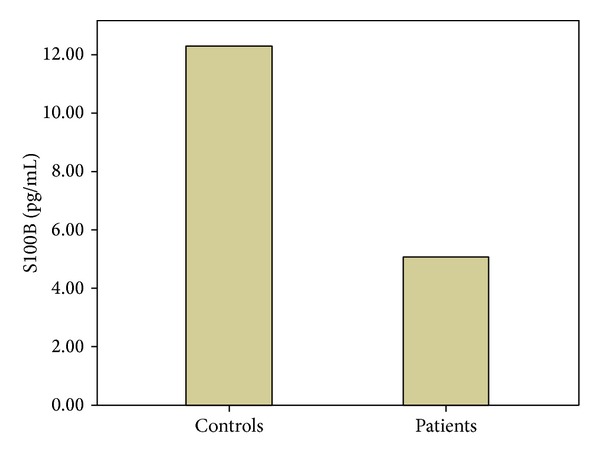
Serum S100B levels in patients with ulcerative colitis and control subjects.

The demographic and laboratory data for the control group and patients with UC are summarized in Table 1. No significant differences were found between the groups in terms of age or sex (*P* > 0.05). BMI was significantly lower in the patients with UC compared with the controls (*P* < 0.05). The ESR and CRP were significantly higher in UC patients than in controls (*P* < 0.001), whereas the WBC was similar in both groups (*P* > 0.05). No GFAP was detected in the serum samples of either UC patients or controls (*P* > 0.05; Table 2). However, we found a significant decrease in the S100B serum levels in patients with UC compared with the controls (*P* < 0.001; Table 2; Figure 1). No correlation between the serum S100B levels and the disease activity or duration was observed (*P* > 0.05). Twenty-four (68.6%) patients were defined as having clinically active disease and 11 (31.4%) patients were in remission. The serum S100B levels did not differ between active and remission UC patients (*P* > 0.05).

## 4. Discussion

The main findings of this study were as follows: (1) GFAP was not detected in either the UC patients or controls, while the serum S100B levels were decreased markedly in the UC patients; (2) there was no correlation between the serum S100B levels and the disease activity or duration in UC patients; and (3) the serum S100B levels were similar in active and remission UC patients.

Similar to astrocytes in the CNS, the EGC release several signaling molecules [17, 18]. S100B protein and GFAP are specific markers of EGC. We hypothesized that disruption of intestinal barrier function or dysregulation of submucosal vascular function could lead to altered serum levels of EGC-derived S100B and GFAP in patients with UC. Mature EGC are rich in the intermediate filament protein GFAP [19]. The expression of GFAP is modulated by glial cell differentiation, inflammation, and injury, and the GFAP level corresponds to the functional state of glial cells [19]. In animals, two classes of glial cells can be distinguished: GFAP-positive and -negative groups [20]. Von Boyen et al. suggested that proinflammatory cytokines control GFAP-positive enteric glia, which are in turn involved in modulating the integrity of the bowel during inflammation [20]. In humans, GFAP expression is altered in the mucosa of patients with IBD [7]. In an animal model of Crohn's disease, the glial network was impaired in noninflamed regions of the intestinal mucosa, which was reflected in a significant decrease in GFAP immunoreactivity [7]. Thus, we might expect GFAP to be detected in the serum of individuals with UC, mainly due to the disruption of EGC populations via the disruption of the peripheral blood-tissue barrier. However, no GFAP was detected in the serum samples of either UC patients or controls. A possible explanation is that GFAP is strictly specific to CNS-related astrocytic processes [21]. Therefore, measuring serum GFAP levels has no utility in patients with UC.

S100B belongs to a multigene family of calcium binding proteins and is expressed abundantly in the brain [22]. It is a small, diffusible neurotrophin that is situated in the cytoplasm or nucleus of cells in both nervous system and non-nervous system tissues [18]. In the brain, as an intracellular regulator, S100B affects protein phosphorylation, energy metabolism, the dynamics of the cytoskeleton constituents, calcium homeostasis, and cell proliferation and differentiation [18, 23]. At the extracellular level, S100B has a dual role: it acts as a neuroprotective or a neurotoxic or neurodegenerative molecule depending on its concentration. At picomolar or nanomolar concentrations, S100B promotes neuron survival, neurite outgrowth, and astrocyte proliferation and negatively regulates astrocytic responses to neurotoxic agents [18, 23]. Micromolar amounts of S100B protein produce neuronal death, as seen in several neuropathologies, such as Alzheimer's disease and Down syndrome [24]. In the human gut, among S100 proteins, only S100B protein is specifically and physiologically expressed by EGC [1, 5, 10], while other members, such as S100A8, S100A9, and S100A12, are found in phagocytes and intestinal epithelial cells in patients with IBD [25]. S100B interacts with target proteins within cells, altering their functions once secreted with the multiligand receptor for advanced glycation end-products (RAGE) [26]. This interaction with RAGE ultimately leads to the transcription of different cytokines, particularly iNOS. Therefore, S100B can be considered an easilydiffusible proinflammatory cytokine that gains access to the extracellular space, especially at sites of immune/inflammatory reaction in the gut [1, 5, 10]. Experimental models have indicated that EGC are able to recognize inflammatory stimuli and that, once activated, they produce and release S100B in up to micromolar concentrations in the intestinal mucosa of patients with UC. They thereby contribute to NO production in the human gut, a hallmark of UC. Moreover, disruption of the peripheral blood-tissue barrier was shown to correlate positively with mucosal inflammation in UC patients, and a high serum level of S100B has been proposed as a marker for BBB disruption following exercise [27]. However, we observed markedly decreased serum levels (pg/mL) of S100B in patients with UC. This contrasts reports of experimental models showing increased S100B expression due to EGC activation [1, 4, 5, 10]. In other words, increased tissue S100B expression was not supported by the increased serum levels. There are some possible explanations for this. One is that its picomolar concentrations may suggest prosurvival effects of this protein in the enteroglial network in UC patients. Another explanation might be that Cirillo et al. [1] included only newly diagnosed UC patients, while the median disease duration was 3 years in our patient group, who were all taking mesalazine and a small number were also taking immunosuppressive agents. These agents might inhibit the inflammatory response related to the high S100B expression in our patients, as described in a previous study, displaying decreases in both the cerebrospinal fluid and serum concentrations of S100B under immunosuppressive treatment in patients with neuroinflammatory pathologies [28]. In animal models of IBD, 5-aminosalicylic acid was found to inhibit the expression of iNOS, which is also involved in the pathogenesis of IBD [29]. It has already been demonstrated that enteroglial-derived S100B protein expression induces NO production in experimental models [1, 4, 5, 10]. Other than the effects of immunosuppressive treatment on NO signaling targeting the vascular endothelium [30], mesalazine treatment could also inhibit iNOS via the inhibition of S100B, which might explain the decreased levels in our UC patients. This raises the question: does mesalazine act via this pathway in the treatment of UC? Alternatively, the persistent inflammation in UC could lead to severe, widespread damage to the enteroglial architecture in UC patients, leading to a reduction in the extracellular secretion of S100B protein. Furthermore, we hypothesized that S100B levels were further decreased in UC patients with high disease activity or duration. However, we failed to identify a relationship between these variables, and larger cohorts are needed to produce more definitive results. In addition, the serum S100B levels did not differ between UC patients with active disease and those in remission. Perhaps their ongoing medical treatment inhibits the EGC-derived S100B release in UC patients with active or inactive disease.

This study had several potential limitations. First, it will be necessary to validate these findings in a larger cohort including untreated UC patients to clarify the exact role of S100B. Second, this study investigated only serum samples; mucosal biopsy analyses should provide additional clarification. Third, data on S100B gene expression profiles, posttranscriptional S100B protein levels determined by western blot analysis, and NO measurements are lacking and might better clarify the mechanisms underlying UC-related enteroglial activation. Fourth, the control group should be well matched regarding BMI, which may be associated with serum S100B levels in humans. Indeed, data on this association are conflicting. Some clinical studies have considered BMI to be an important confounding factor for examining the role of S100B protein [31–33], while others have not [34, 35].

In conclusion, this is the first study to demonstrate that, unlike the markedly increased expression of GFAP and S100B observed in experimental models, S100B levels were decreased significantly in patients' serum samples. After confirmation achieved by the inclusion of serum from the untreated UC patients in a larger cohort, S100B could be suggested to have an occult role, whereas GFAP might be of no diagnostic value in patients with UC. Despite the abovementioned limitations, we consider this to be a pioneer study that may guide subsequent research on this issue. Indeed, we wish to validate the present findings in a larger cohort, with additional research on NO measurements and the inclusion of untreated patients, all of which we lacked due to limited funding. Future large-scale longitudinal studies that overcome the current study's limitations will present a more detailed view of the underlying mechanisms of the regulation of S100B and its actual role as a noninvasive biomarker of EGC activation in patients with UC.

## Figures and Tables

**Table 1:** Demographic and laboratory data of patients with ulcerative colitis and controls.

Variables	Patients (*n* = 35)	Controls (*n* = 40)	*P*
Age (years)	44.22 ± 13.34	48.92 ± 9.16	0.077
Gender (male/female)	23 (65.7)/12 (34.3)	19 (47.5)/21 (52.5)	0.786
BMI (kg/m^2^)	29 ± 12	33 ± 12	0.001
ESR (mm/h)	30.62 ± 24.54	15.77 ± 11.10	<0.001
CRP (mg/dL)	10.3 (4.8–30.2)	0.45 (0.3–0.97)	<0.001
WBC (10^3^/mm^3^)	7.80 (6.45–9.36)	7.55 (6.82–9.20)	0.962
Disease duration (years)	3 (1–10)	—	—

Values are expressed as *n* (%), mean ± SD, or median (25th–75th percentiles). BMI; body mass index; ESR: erythrocyte sedimentation rate; CRP: C-reactive protein; WBC: white blood cells.

**Table 2:** Serum levels of GFAP and S100B in patients with ulcerative colitis and controls.

Variables	Patients (*n* = 35)	Controls (*n* = 40)	*P *
GFAP (ng/mL)	0 (100)	0 (100)	0.895
S100B (pg/mL)	4.54 (4.0–5.31)	12.5 (10.32–19.8)	<0.001

Values are expressed as *n* (%) or median (25th–75th percentiles). GFAP indicates glial fibrillary acidic protein.
